# A Multimodal Approach for Real Time Recognition of Engagement towards Adaptive Serious Games for Health

**DOI:** 10.3390/s22072472

**Published:** 2022-03-23

**Authors:** Konstantinos Mitsis, Konstantia Zarkogianni, Eleftherios Kalafatis, Kalliopi Dalakleidi, Amyn Jaafar, Georgios Mourkousis, Konstantina S. Nikita

**Affiliations:** 1 School of Electrical and Computer Engineering, National Technical University of Athens, 15780 Athens, Greece; kmhtshs@biosim.ntua.gr (K.M.); kzarkogianni@biosim.ntua.gr (K.Z.); leftkal@biosim.ntua.gr (E.K.); kdalakleidi@biosim.ntua.gr (K.D.); 2 Biomedial Simulations and Imaging Laboratory, National Technical University of Athens, 15780 Athens, Greece; amyn.jaafar@outlook.fr (A.J.); mourkousis@gmail.com (G.M.)

**Keywords:** serious games, health, adaptive, procedural content generation, sensors, real time recognition, engagement

## Abstract

In this article, an unobtrusive and affordable sensor-based multimodal approach for real time recognition of engagement in serious games (SGs) for health is presented. This approach aims to achieve individualization in SGs that promote self-health management. The feasibility of the proposed approach was investigated by designing and implementing an experimental process focusing on real time recognition of engagement. Twenty-six participants were recruited and engaged in sessions with a SG that promotes food and nutrition literacy. Data were collected during play from a heart rate sensor, a smart chair, and in-game metrics. Perceived engagement, as an approximation to the ground truth, was annotated continuously by participants. An additional group of six participants were recruited for smart chair calibration purposes. The analysis was conducted in two directions, firstly investigating associations between identified sitting postures and perceived engagement, and secondly evaluating the predictive capacity of features extracted from the multitude of sources towards the ground truth. The results demonstrate significant associations and predictive capacity from all investigated sources, with a multimodal feature combination displaying superiority over unimodal features. These results advocate for the feasibility of real time recognition of engagement in adaptive serious games for health by using the presented approach.

## 1. Introduction

Serious games (SG) for health have been a topic of growing attention in recent years. According to one of the most widely accepted definitions, SGs are games designed for a primary purpose other than pure entertainment [1]. SGs can provide effective means for addressing several health-related challenges such as the training of health professionals, raising awareness, rehabilitation, disease monitoring and diagnosis, the promotion of behavioral lifestyle changes, and the management of mental health [2,3]. However, despite recent advances in the field, limited research has been conducted on tailoring persuasive game design to specific players’ needs [4]. The reported results demonstrate differences in receptivity of persuasive strategies in SGs for health, among multiple user types, indicating that intuitive, one-size-fits-all design approaches are not always effective. In addition, ambiguous results have been reported regarding the learning effectiveness of SGs, thus, further motivating research into enhancing game adaptivity [5]. A recent review study [6] highlights the importance of delivering personalized content in SGs and employs the term “individualization” for this purpose. Individualization in SGs can not only enhance the game experience, but also address specific user needs linked to the game’s serious purpose, like task performance or knowledge acquisition.

Lately, novel technologies are increasingly being employed to develop individualized SGs [7]. SGs can greatly benefit from procedural content generation (PCG) techniques, a term used to describe methodologies that generate game content either automatically or with minimal guidance [8]. Advancements in the fields of data analysis and artificial intelligence, along with the development of low-cost, portable, and unobtrusive sensors, enable real-time individualization of PCG based on data collected from a multitude of sources [9]. PCG methods can be augmented through real-time recognition and employment of engagement in a constant feedback loop that adapts game content based on the player state [7,10]. Engagement has been argued to be an essential element of the game experience [11], related to positive and negative affects through game activities and the accomplishment of objectives. Different perspectives have been identified regarding the construct and measurement of engagement according to a recent review study [12]; the study adopts a three-part framework for engagement that includes the dimensions of behaviour, cognition, and affect. Recognition of the affective aspect of engagement and its employment in an “emotion-sensitive adaptive game approach” [5] can lead to heightened task persistence and an improved learning process. There are various sources from which to collect informative data regarding players’ affective states during game play, such as self-reporting (e.g., game experience questionnaires), in-game metrics, wearable sensors (e.g., electrocardiogram (ECG), electroencephalogram (EEG) electromyogram (EMG), electro-dermal activity sensors), and posture recognition sensors [12,13]. More specifically, pressure sensors, for posture and mobility monitoring, have been employed for this reason in learning environments [14], or during intense cognitive activity [15]. Features extracted from heart rate sensors have been identified as potential detectors of affective states, stress, and learning [16,17]. Data collected from sensors can be augmented by in-game metrics and analytics that exhibit promising associations with the learner’s engagement [18,19]. Labelling engagement during high cognitive function, such as learning, is considered a difficult task and various approaches and methods have been proposed to address it [20]. One of the main limitations reported in recent literature is the assessment of the dynamic nature of engagement, as the most prominent annotation tools, like self-reporting engagement scales, produce as little as one label for entire interaction sessions [21,22]. This is particularly hindering for the training of ML techniques for the recognition of engagement in real time, as transient changes in engagement are not captured.

Focusing on the health sector, the rapid advancements in sensing technologies make feasible the implementation of patient-tailored interventions supported by properly designed SGs. More specifically, sensor-based adaptive SGs, including player profiling, in terms of health status and lifestyle habits, along with recognition of engagement in real time, have the capacity to address important challenges in chronic disease management such as the presence of inter- and intra-patient variability while offering low-cost services at the point of care. Research on the recognition and employment of engagement and other affective states to achieve individualization in SGs for health is still limited. A handful of relevant publications have been identified, with the most common case being biofeedback SGs for stress management. Features extracted from breathing signal and heart rate variability (HRV) analysis have been used to predict affective states, such as stress and engagement during game play, in a biofeedback context for stress management therapy [23,24]. The use of ECG signal transmitted in real time to a therapist has also been reported in the context of a virtual reality SG for emotional regulation in adolescents [25]. Moreover, a methodology for multimodal affect recognition for SGs targeting the treatment of behavioural and mental disorders and chronic pain rehabilitation has been presented [26]. A SG for automated personalised exposure therapy that includes experience-driven PCG has also been proposed, employing machine learning (ML) techniques to predict stress from physiological signals [27]. Additionally, emotion recognition has been applied on speech components to support SGs aimed towards cognitive-based treatment for mental disorders, with results indicating the successful recognition of interest, boredom, and anger [28]. However, the identified approaches are related to SG interventions targeting mainly mental health and disorders. Additionally, limited progress has been reported towards transferring recent advancements in sensing and PCG techniques to further enhance SGs as healthcare interventions and tackle modern problems with implications in the self-health management of chronic conditions, such as increased sedentary time [29].

Novel technologies, from the fields of ML and deep learning, utilizing data from various sources, have been applied to enhance PCG in entertainment games [30]. For instance, a recently proposed PCG framework employs intrinsically motivated reinforcement learning that builds knowledge about the player’s preferences by searching for unexplored information and being rewarded for discoveries [31]. Intrinsically motivated reinforcement learning, thus, makes feasible the development of experience-driven PCG that considers the impact of the generated content on the player’s affective state. Such frameworks can be combined with novel techniques that procedurally generate individualized content specific to self-health management needs and preferences [32]. The procedural generation of SG content, built to accommodate educational and behavioural objectives regarding the targeted condition, is thus controlled by an engagement feedback loop. These objectives include, amongst others, knowledge about the management of the condition and daily self-health management goals [33]. Maximizing player engagement not only promotes the SG’s effectiveness towards these objectives, but also increases adherence to the intervention, leading to sustainable improvement in self-health management. Additionally, sensors employed for the recognition of engagement can produce clinically relevant data or lifestyle parameters [34,35]. The integration of such data in the SG feedback loop, along with in-game metrics, can ultimately lead to PCG that individualizes SG content according to condition and player specific needs while promoting adherence through engagement. 

The purpose of the current study is to investigate the feasibility of such frameworks through the conduct of a carefully designed experimental process involving the interaction of volunteers with a custom SG for the promotion of nutrition and food literacy. The employed SG has been selected for two reasons, firstly due to its capability to serve as an intervention in self-health management for chronic conditions, and secondly, due to its potential to incorporate the procedural generation of SG content related to the targeted condition. During the experiment, heterogenous data from a multitude of affordable and unobtrusive sensors and in-game metrics have been collected to provide insight regarding a multimodal approach in recognition of engagement. The predictive power of features extracted from the collected data in terms of real time recognition of engagement has been assessed through a detailed analysis. An approximation of the ground truth has been produced through the self-annotation of perceived engagement in a continuous manner by leveraging a state-of-the-art tool designed for affective recognition in video games. By generating annotation traces of perceived engagement during actual gameplay, the present study introduces an effective combination amongst the investigated data sources towards real-time recognition of engagement. Through the employment of a suitable SG as a case study, the proposed approach investigates the potential of PCG in adaptive SG interventions for chronic health conditions.

## 2. Materials and Methods

**Figure 1:**
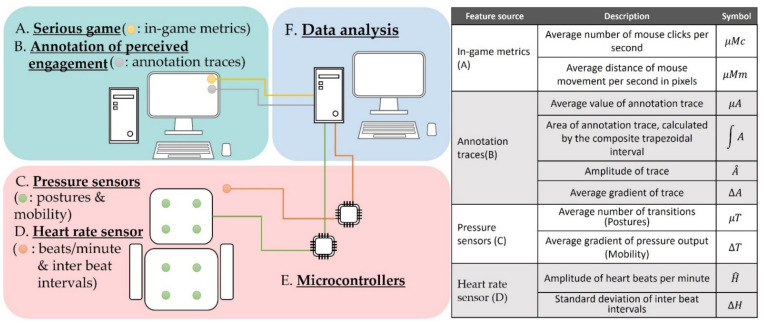
Setup of the experimental process and summary of extracted features.

**Figure 2:**
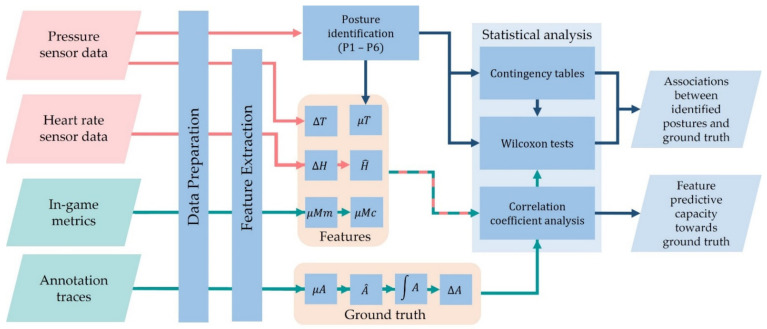
Flowchart for data analysis. (Features and ground truth explained in Figure 1).

The setup of the experimental process is presented in Figure 1. It includes three interconnected spaces enabling the necessary data acquisition and analysis. In-game metrics are gathered through the player’s interaction with the SG while annotation traces, to be considered as the ground-truth level of engagement as perceived by the player during game play, are generated through an annotation tool. Sensing data are collected by means of: (i) pressure sensors placed on a chair for identifying postures and mobility, and (ii) a heart rate sensor providing the heart rate (beats per minute) and inter-beat intervals. Two microcontrollers are employed for the acquisition of sensor data. The collected heterogeneous data feed the data analysis space in order for the latter to apply thorough statistical analysis, investigating the potential of sensor and in-game metrics data towards real-time engagement recognition. A summary of the investigated features is presented in Figure 1, while the data flow and the adopted approach is described in Figure 2. Each space is explained in detail in the following sections.

### 2.1. User Interaction Space

#### 2.1.1. Serious Game

“Express Cooking Train” (ECT) [36] is a SG which has been developed with GameMaker Studio 1 [37] for Microsoft Windows and has been employed as a case study for the experimental process. ECT employs ontology modelling to create a gamified virtual kitchen environment and provides a safe trial and error simulation environment to support its educational goals towards healthier cooking practices and eating habits. Ontology modelling facilitates the incorporation of PCG techniques that control SG content. The SG is built on a theoretical conceptual framework that incorporates various game mechanics and reward systems [36], with the goal of empowering and supporting sustainable healthy lifestyle changes.

Preliminary studies have proved ECT to be equally effective as a traditional educational intervention, while achieving high levels of user acceptance [38]. The game is played in missions, with the player taking control of a train trying to reach the next destination. ECT is set in a post-apocalyptic setting and during these short trips, huge monsters chase the train. The player must cook healthy meals in the kitchen wagon and launch them with a catapult towards the monsters to satisfy their appetite and reach the next train station safely. Adding to the game difficulty, the train inventory contains mostly junk food recipes, forcing the player to explore ways of including healthier ingredients and applying better meal preparation techniques, as the monsters grow angry if junk food is thrown their way.

**Figure 3:**
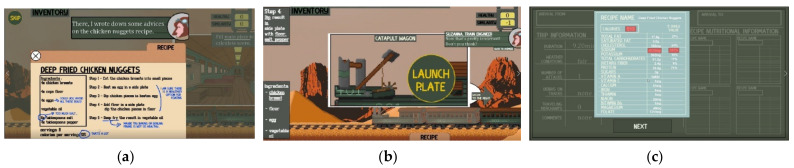
Screenshots from the three phases of Mission-1: (**a**) Tutorial; (**b**) Gameplay; (**c**) Review.

During the experimental process, a version of ECT that contains three game missions is deployed. Mission-1 includes a tutorial phase (Figure 3a), a gameplay phase (Figure 3b), and a review phase (Figure 3c). During the tutorial phase, players are given instructions about the game interface and mechanics, as they are introduced to the game world and objectives. Interaction during the tutorial is quite minimal as players are presented with explanatory text boxes and experiment with game functionalities under guidance. As the tutorial ends, a hungry monster appears and chases the train, signifying the beginning of the gameplay phase. During this phase, players apply knowledge acquired in the tutorial to prepare healthy meals for the chasing monster and avoid it until the train reaches its destination. Finally, the review phase launches with a small cinematic of the train escaping the monster in the case of a successful mission, or the monster catching up with the train in the case of defeat. Following the cinematic, a review screen appears, containing game statistics, nutritional facts for the recipes used, new discoveries, and unlocked achievements. After Mission-1 is complete, players can continue playing for a maximum of two additional missions. The additional missions feature small train trips and thus include shorter gameplay and review phases.

During playthroughs, the SG monitors in-game metrics, including the number of mouse clicks, mouse click duration, and mouse idleness. Additionally, game score, mission progress and in-game decisions, cooking simulation parameters, and game events are collected. Preliminary analysis of mouse-related user interaction data has been presented in [38], with findings indicating that participants with high and low interaction, as indicated by average clicks per second, have scored significantly higher in positive game experience scales of the game experience questionnaire, in comparison to participants with intermediate levels of interaction. In the present study, two features based on mouse interaction have been extracted, namely average clicks per second (μMc) and average mouse movement (μMm), as they have been reported to provide measures of cognitive function and engagement [13]. Mouse movement measures the cursor distance travelled per second, in pixels.

#### 2.1.2. Annotation of Engagement

**Figure 4:**
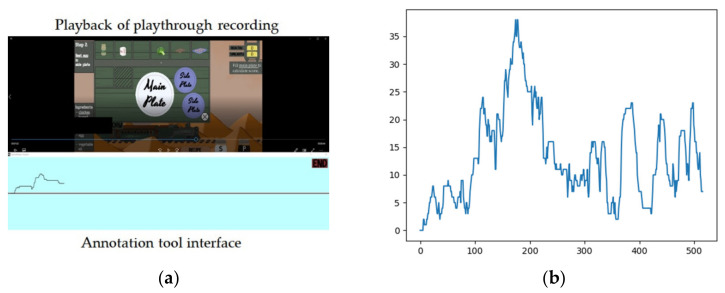
(**a**) The interface of the annotation tool along with the playthrough recording as shown in an annotation session; (**b**) Example of an annotation trace of perceived engagement.

To capture dynamic changes in engagement in real time during play, an annotation tool was employed for the approximation of the ground truth regarding player engagement. The tool (Figure 4a), created with GameMaker Studio, is based on the design of RankTrace [39] that allows for continuous and unbounded annotation of affect while the player is being presented with screen recordings of playthroughs. Through this approach, players generate a continuous annotation trace of perceived engagement immediately after their playthrough. Annotation values were produced through the mouse wheel, with one annotation sample being collected per second. Sampling frequency was selected to facilitate the harmonization of the heterogeneous data sources, since annotation traces were used as a reference along with playthrough recordings. An example of an annotated trace is shown in Figure 4b. Annotation data were normalized in the range [0, 1] using the minimum and maximum values of each individual annotation trace. From each observation frame generated, four statistical features [39] were extracted: the mean annotation value (μA); the area of the annotation trace (∫A), calculated by the composite trapezoidal integral and normalized by duration; the amplitude $\left(\hat{A}\right)$, calculated by the difference between maximum and minimum value; and the average gradient of the annotation trace $\left(\Delta A\right)$.

### 2.2. Sensing Space

**Figure 5:**
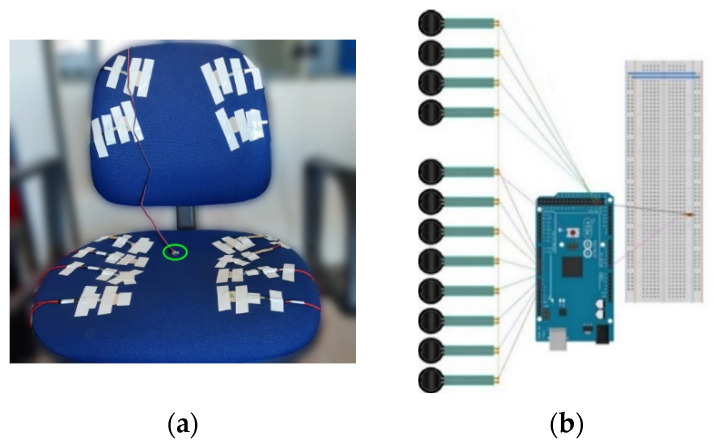
(**a**) Smart chair with initial arrangement of 12 pressure sensors. PulseSensor (placed in the user’s ear lobe) is marked by the green circle; (**b**) Microcontroller setup for acquisition of data from the smart chair.

Sensor measurements were acquired through two Arduino Mega 2560 R3 [40] microcontrollers, one for the pressure sensors (Figure 5b) and one for the heart rate sensor, following the setup used in [41]. The microcontrollers transferred data to a desktop computer through a USB interface. PC port control was provided by the Python 3.6.5. The user interface to control the sensors was also developed in Python. A case was crafted and affixed to the back of the smart chair to hold the microcontrollers and breadboards in place and facilitate cable management. 

#### 2.2.1. Smart Chair

In the current study, a smart chair was employed to identify sitting postures and monitor their variations. A set of pressure sensors, FSR101 Shuntmode from Sensitronics [42], were placed on the seat and back of an office chair to measure pressure exerted by body weight during playthroughs of the SG on a desktop computer. The sensors were strapped on the chair, along with the cables linked to them. Afterwards, two cloth covers were fastened on the chair, one on the seat and one on the back, to secure sensor placement while reducing the risk of bias by making sure that sensors’ location is not visible to the participants. Measurements were recorded from all sensors, with no load on the chair, to ensure that pressure from the tape or cloth cover did not affect sensor output. Eight sensors were placed to monitor pressure distribution on the seat of the chair (four under each thigh), while four sensors were placed on the back of the chair (two sensors on each side) to detect sitting back postures. Sensor arrangement along with the employed smart office chair are shown in Figure 5a.

Data collected from the smart chair were used to identify sitting postures during playthroughs based on a sensor activation methodology [15]. Postures were identified by detecting different sensor activation patterns and matching them to predefined sitting positions. A sensor placed on the smart chair was considered active when its output value exceeded a certain threshold. A set of six sitting postures (Table 1) were identified during the experimental process. Participants were observed to assume mainly upright postures, always activating most of the four front seat sensors due to the placement of monitor, mouse, and keyboard on the office desk. Additionally, no postures including leg crossing were observed during playthroughs. The data collected supported these observations, with sensors situated in the middle of the seat always being active. Based on these observations and preliminary analysis, data acquired from sensors 1, 2, 5, and 6 (Figure 5a, Table 1) were excluded from posture identification and the activation patterns shown in Table 1 were selected. Postures P1 and P4–P6 included activated sensors on the back of the chair, whereas postures P2 and P3 did not. Two features were extracted from the observation frames. First, the total number of posture transitions (μΤ), normalized by duration was extracted to acquire a macroscopic measure of participant mobility [15]. Secondly, a feature of relative change (ΔΤ), calculated as the average gradient in sensor output, was extracted from the sensors included in posture detection, to provide insight regarding mobility observed in pressure distribution [14].

The sensor activation threshold constitutes a vital component for the reliable identification of sitting postures. In this study, a separate small-scale experiment was conducted to estimate a general activation threshold. A group of six individuals within the healthy BMI range (BMI: 18.5–25) were recruited, yet these participants were excluded from the main experimental process to reduce the risk of bias. After an initial visual presentation of postures P1–P6, participants were told to test them, while sitting on the smart chair, with no time limit. Consequently, these postures were displayed through an application developed in GameMaker Studio, for 10 s each in random order, until all possible posture transitions had been presented. Participants were instructed to assume postures as they appeared on the screen.

**Figure 6:**
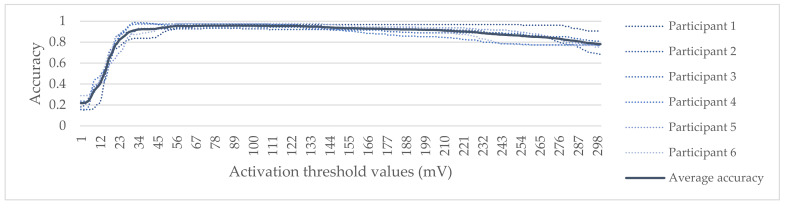
Accuracy results towards the determination of sensor activation threshold for posture identification.

To determine the appropriate threshold, a wide range of sensor output values (1–300 mV) were considered to identify sitting postures. The obtained average accuracy across all participants for all activation thresholds is depicted in Figure 6. The maximum average accuracy (0.96) was achieved for activation threshold values ranging from 86 mV to 93 mV. Multiple ANOVA single factor tests were applied on batches of 50 consequent activation threshold values, to investigate statistically significant differences in mean accuracy values across participants with respect to different threshold values. No significant differences were observed for activation threshold values higher than 30 mV (*p* > 0.05). Consequently, the general activation threshold selected for sitting posture identification was 90 mV.

#### 2.2.2. Heart Rate Sensor

The heart rate sensor employed within the frame of this study is the PulseSensor from World Famous Electronics LLC (New York, NY, USA) [43]. PulseSensor is an affordable and non-obtrusive sensor that can be placed around the finger, or on the ear lobe. For this study, the sensor was placed on the ear lobe to avoid obstruction during game play. The sensor detects pulses through a light-emitting diode generating a photoplethysmography (PPG). Inter beat intervals (ms) along with beats per minute were obtained in real time from PPG. Data from the PulseSensor were collected at a rate of approximately 25 Hz. Inter-beat intervals were isolated and ranked in time order. The intervals were then preprocessed for the removal of ectopic beats and outliers in Python 3.6.5. From the resulting values, two features were extracted: the amplitude of heart beats per minute ($\hat{H}$), measured as the difference between maximum and minimum heart rate value, and the standard deviation (σH) of inter-beat intervals [44]. 

### 2.3. Participants and Experimental Protocol 

A total of 26 participants, 19 male and 7 female, aged 26.2 ± 4.6, mostly undergraduate and postgraduate students at the National Technical University of Athens (NTUA), were recruited. No participant had any apparent mobility or visual impairment and most participants had normal BMI scores (BMI score: 18.5–25), except for two slightly overweight (BMI score: 25–27), and two slightly obese (BMI score: 30–35). As ECT is in English, all participants reported a good understanding of the English language.

Upon arrival, participants were given a brief description of the experimental process, the aim of the study and the potential outcomes, while being encouraged to engage in conversation about possible concerns. Subsequently, they were provided with consent forms that included a detailed description of the experimental process. All participants provided written informed consent and the study was approved by the Ethics Committee of NTUA. Upon providing their consent, the participants sat on the smart chair in front of a desktop computer and were asked to assume a comfortable position. The heart rate sensor was then placed on the ear lobe of their choice, and they were asked to perform exploratory movements while seated, to confirm that the sensor is not hampering them in any way. After setup was completed and participants felt comfortable, they were instructed to fill out some digital questionnaires including information about demographics and their exposure to gaming and cooking habits [38]. Participants were then instructed to start Mission-1 at their leisure while being given the option to play the two additional game missions. Once the playthrough session was completed, the participants were given instructions on how to use the annotation tool. After a short time in which to familiarize themselves with the mouse wheel control, participants annotated their perceived engagement on the video playback of their game session and concluded their participation. 

Mission-1 was played by all participants, while Mission-2 and Mission-3 were played by 14 and 3 participants, respectively. 

### 2.4. Data Analysis Space

#### 2.4.1. Data Preparation

Data harmonization was conducted to ensure the synchronization of heterogeneous data collected from different sources during the experimental procedure. The considered data sampling rate for in-game metrics and pressure sensing data was 1 Hz. Annotation traces and recordings of playthroughs were employed as synchronization reference. A moving average filter with a cutoff frequency of 1 Hz was applied on the signals obtained from the pressure sensors for noise removal and synchronization. Data from the heart sensor were preprocessed and synchronized as described in Section 2.2.2.

**Figure 7:**
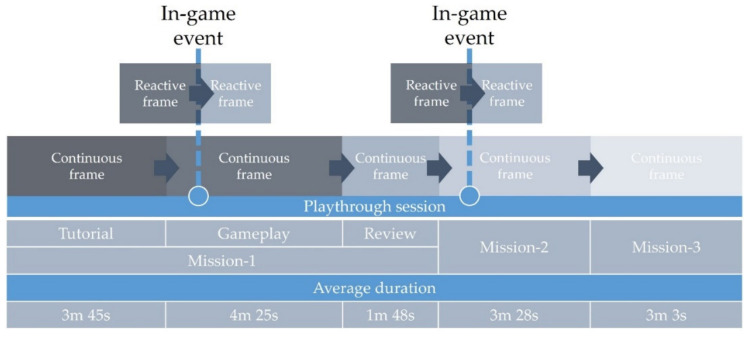
Continuous and reactive frames.

Different types of observation frames (continuous and reactive) were considered [39] to link the ground truth, investigated features, and identified postures with different types of gameplay and specific game mechanics, as depicted in Figure 7. Initially, continuous, and non-overlapping observation frames, representing different game phases (Tutorial, Gameplay, Review, Mission-2, Mission-3) were generated. The average duration of all game phases for all participants is presented in Figure 7. Due to the limited number of participants advancing to the Mission-3, the frames corresponding to this mission were omitted from the analysis. Game phases correspond to the different types of gameplay, with engagement levels expected to vary according to user preferences. The Tutorial phase is a linear and educational phase, with rich text content and minimal interaction (Figure 3a). The Gameplay phase requires a higher degree of game interaction and provides an exploration experience, with the player being free to experiment with ingredients and cooking tools while switching between train wagons (Figure 3b). Furthermore, the Gameplay phase includes the danger imposed by the chasing monster and the possibility of defeat. The Review phase contains a lot of information yet allows the player to survey it freely (Figure 3c). Additionally, the Review phase features the element of reward in the form of score points, discoveries, and achievements unlocked. The Mission-2 phase provides a similar gameplay experience to the one provided by the Gameplay phase. 

Reactive observation frames were specified as those triggered by in-game events tied to specific game mechanics. These events include a visual alarm indicating danger and triggered by close monster proximity to the train, and monster-related player interaction such as launching a meal to the monster with the catapult or clicking on the monster. These events were selected to point towards game moments that favor changes in player engagement. The nature of these events is memorable, aiming to produce more accurate annotation traces of perceived engagement around them. Furthermore, the manifestation of these events is not scripted and is based on the player’s actions and performance; hence players cannot expect or plan them, thus reducing the risk of bias. Each event generates two reactive observation frames, prior to and after the event. A total of 89 in-game events were produced during the participants’ playthroughs. Reactive frames of different duration, 10 s and 30 s, were investigated in accordance with current practice for ultra-short analysis of heart rate variability [45,46].

#### 2.4.2. Statistical Data Analysis

Features were extracted from all data sources and sitting postures were identified for all observation frames across participants. The feasibility of real time recognition of engagement during play was investigated in two parts. The first part relied on statistical analysis of sitting postures and features of perceived engagement as extracted from annotation trace. Contingency tables were generated for each observation frame, continuous and reactive, to perform transition analysis [15]. The element (P_x_, P_y_) of the contingency table represents the number of times a transition was identified from posture P_x_ to posture P_y_. Distributions of identified postures were extracted from the contingency tables for each observation frame. Wilcoxon signed-rank tests were employed to search for statistically significant differences between observation frames in terms of identified postures and perceived engagement. Additionally, whisker boxplots were created from the annotation features to accurately present trends in perceived engagement. 

The second part of the analysis evaluates the predictive capability of features extracted from sensors and in-game metrics, based on relative changes observed between adjacent observation frames, towards perceived engagement. To this end, an analysis based on correlation coefficients [39,47] was conducted for continuous observation frames and reactive observation frames, separately. More specifically, a correlation coefficient,

(1) ci−jz=∑k=1Nzk,i,jN, with i∈μA, ∫A, A^, ΔA, j∈μMc, μMm, μΤ, ΔT, H^, σH

was calculated for every possible combination of pairs between annotation features (*i*), and sensor and in-game metrics features (*j*). For each participant, the observation frames were ranked in order of time, with *N* representing the total number of adjacent frames across all participants. By measuring agreement in relative change in features *i* and *j* between the *k*-th pair of adjacent frames, $z_{k}^{i,j}$ was calculated as,

(2) $$z_{k}^{i,j}=+1,\;if\;relative\;change\;\mathrm{of}\;i\;\mathrm{and}\;j\;\mathrm{match}\;z_{k}^{i,j}=-1,\;if\;relative\;change\;\mathrm{of}\;i\;\mathrm{and}\;j\;\mathrm{does}\;\mathrm{not}\;\mathrm{match}$$

If clear relative change in any of the examined features was not present, the corresponding $z_{k}^{i,j}$ was not included in the calculation of $c_{i-j}\left(z\right)$. The average number of pairwise comparisons (*N*) of all investigated feature pairs for all participants, per type of observation frame, after the exclusion of pairs that did not display clear relative change, is shown in Table 2. Relative change in the average number of posture transitions (μΤ) was clear in very few comparisons (25.2 ± 13.6) and no statistically significant resulting values of $c_{i-\mu Τ}\left(z\right)$ were observed. As such, $c_{i-\mu Τ}\left(z\right)\;\mathrm{values}$ were excluded from the corresponding analysis. The *p*-values of $c\left(z\right)$ were calculated through the binomial distribution, with correlation being highly significant for p<1%, and significant for 1%<p<5%. 

Motivated by the superior performance that can be achieved through combining different modalities [48], a majority voting scheme was investigated towards the generation of a new multimodal feature (*V*). The choice of this particular combination scheme was based on its approved robustness in binary cases [49]. The majority voting scheme assumes the dominant relative change observed in all primary (sensors and in-game metrics) features. Voting includes only clear relative changes, and in case majority voting does not produce a clear result, the pair is excluded from the calculation (Table 2). Consequently, $c_{i-V}\left(z\right)$ is calculated for all features (*i*) of perceived engagement.

## 3. Results

Results from both parts of the data analysis are presented for the investigated continuous and reactive observation frames. Data collected from two participants were excluded from both parts of the analysis for reactive observation frames, as in-game events were not recorded properly by the SG. Additionally, data collected from two more participants were excluded from the second part of the analysis due to movement of the heart rate sensor during play. 

**Figure 8:**
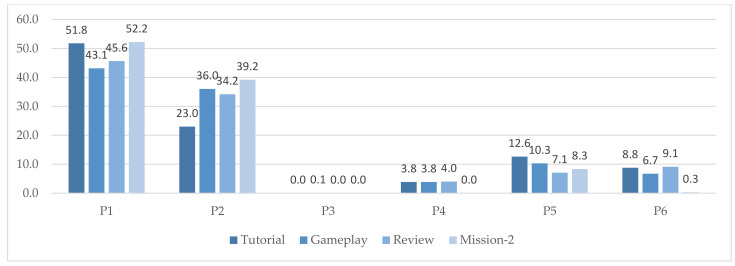
Distribution of postures identified for Tutorial, Gameplay, Review, and Mission-2.

The distribution of identified postures (P1–P6) for continuous observation frames, Tutorial, Gameplay, Review, and Mission-2 is depicted in Figure 8. A statistically significant decrease in the percentage of postures including the back of the chair was observed from Tutorial to Gameplay (one-sided Wilcoxon: *p* = 0.03). Posture P3 (front sitting) was identified in very few occasions (≤0.1%) across all collected data. No other statistically significant changes in identified postures were identified between continuous observation frames.

**Figure 9:**
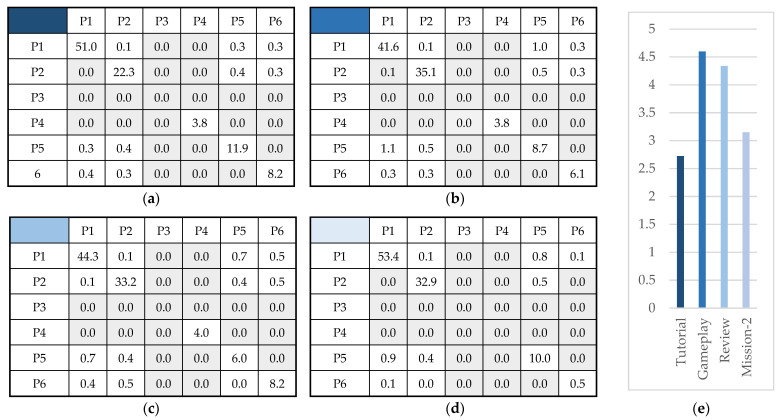
Contingency tables for continuous frames: (**a**) Tutorial; (**b**) Gameplay; (**c**) Review; (**d**) Mission-2; and (**e**) Percentage of posture transitions identified in each frame.

Contingency tables showing the percentages of postures for continuous observation frames are presented in Figure 9a–d. The percentage of transitions is presented in Figure 9e, with participants demonstrating the highest mobility in Gameplay. However, no statistically significant changes were present between any pairs of observation frames. 

**Figure 10:**
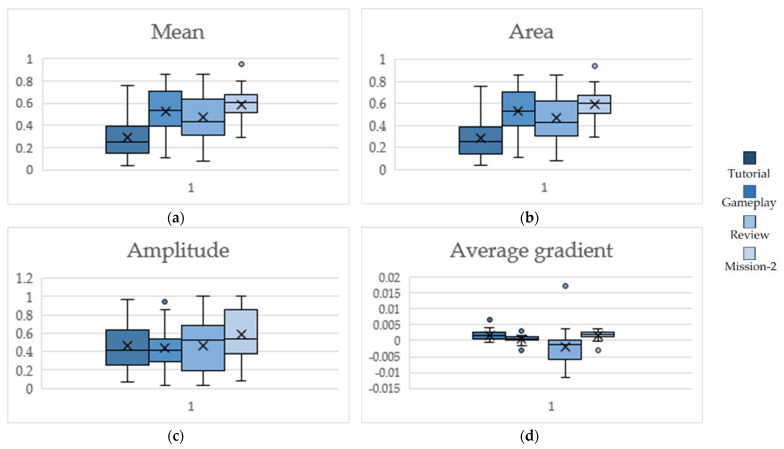
Whisker boxplots for Tutorial, Gameplay, Review and Mission-2, for (**a**) mean value of perceived engagement, (**b**) area of annotation trace, (**c**) amplitude of engagement, (**d**) average gradient of engagement.

Whisker boxplots for features extracted from the annotation traces of perceived engagement are presented in Figure 10. The two-side Wilcoxon test revealed a statistically significant (*p*-value < 0.01) increase of 82.75% and 79.31% from Tutorial to Gameplay in mean value (μA) (Figure 10a) and area of the annotation trace (∫A) (Figure 10b), respectively. A statistically significant increase, of 34.09% and 37.20%, was also present from Review to Mission-2 for these two features, respectively. The decrease depicted for μA and ∫A from Gameplay to Review was not significant. Changes observed in amplitude ($\hat{A}$) (Figure 10c) were not statistically important. A decrease of 76.47% and 428.79% in the average gradient of the annotation trace (ΔA) (Figure 10d), from Tutorial to Gameplay and from Gameplay to Review, respectively, were found to be statistically significant (two-side Wilcoxon, *p* < 0.01).

**Figure 11:**
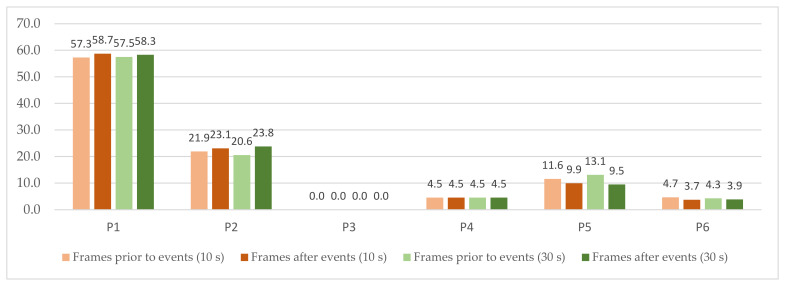
Distribution of postures identified during reactive frames.

The above presented analysis was also applied for reactive observation frames. The distribution of identified postures (P1–P6), for reactive frames of 10 and 30 s, is depicted in Figure 11. A statistically significant decrease in postures including the back of the chair was present in 30 s frames (two-side Wilcoxon: *p* = 0.03). No other statistically significant change in postures between reactive observation frames was observed.

**Figure 12:**
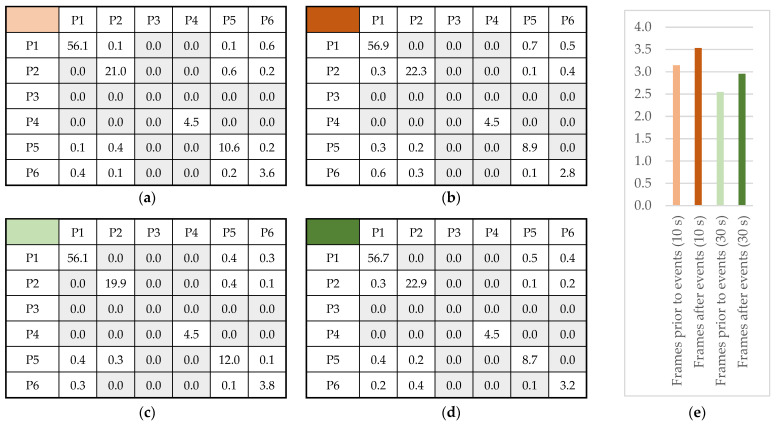
Contingency tables for reactive frames: (**a**) Frame prior to event (10 s); (**b**) Frame after event (10 s); (**c**) Frame prior to event (30 s); (**d**) Frame after event (30 s); and (**e**) Percentage of posture transitions identified in each frame.

**Figure 13:**
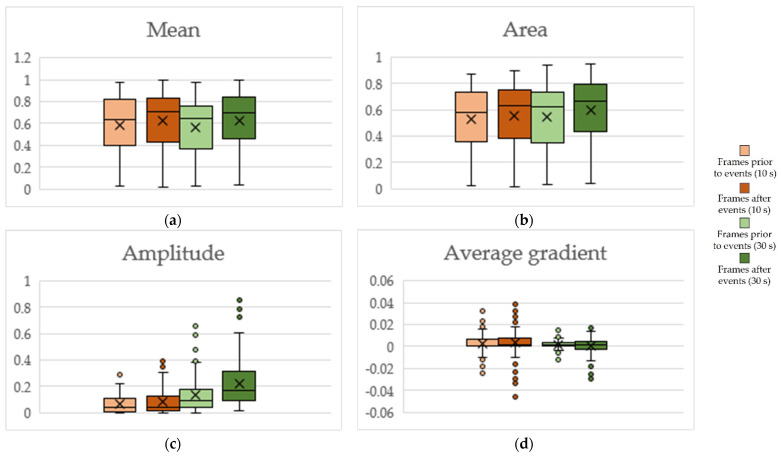
Whisker boxplots for reactive frames, for (**a**) mean value of perceived engagement, (**b**) area of annotation trace, (**c**) amplitude of engagement, (**d**) average gradient of engagement.

Contingency tables including the percentages of postures for reactive observation frames are presented in Figure 12a–d. The percentage of transitions is presented in Figure 12e, with participants demonstrating higher seated mobility in frames after in-game events for both investigated frame durations. However, no statistically significant changes were identified in both cases. Whisker boxplots for features extracted from the annotation traces of perceived engagement for reactive frames are presented in Figure 13. 

A statistically significant increase of +5.6% and +5.5% was observed in μA (Figure 13a) and ∫A (Figure 13b), respectively (two-sided Wilcoxon: *p* < 0.01), for 10 s observation reactive frames. The corresponding increases for 30 s frames were up to +10.4% and +9.5%, respectively (two-sided Wilcoxon: *p* < 0.01). A significant increase in $\hat{A}$ (Figure 13c) was also present in 30 s observation reactive frames (two-sided Wilcoxon: *p* < 0.01). Changes observed in average gradient (Figure 13d) were not found to be statistically significant.

For the second part of the analysis, the predictive capability of sensor and in-game features (Figure 1), along with the multimodal feature *V*, towards features of perceived engagement are presented in Table 3. The amplitude $\left(\hat{A}\right)$ of the annotation trace presents the most cases of statistically significant correlation with sensor and in-game metrics features, thus highlighting its capacity to represent the hypothesized ground truth independently of type and duration of the observation frame. In particular, a negative correlation was observed with average mouse clicks (μMc) and variability of inter-beat intervals (ΔH) for 10 s reactive frames. Additionally, significant, and highly significant positive correlations were observed with the amplitude of heart beats per minute ($\hat{H}$), and voting (*V*) for both 30 s reactive and continuous frames. Finally, a highly significant positive correlation with mouse movement (μMm) and a significant correlation with the average gradient of pressure sensors (ΔΤ) were evident in 30 s reactive frames. The mean value (μA) and the area (∫A) of the annotation trace were correlated significantly with *V*, in 10 s and 30 s reactive frames, with both in-game metrics features in 30 s reactive frames, and ΔΤ in 10 s reactive frames. The average gradient of the annotation trace (ΔA) did not display significant correlations with any sensor or in-game metrics feature.

## 4. Discussion

Results from the first part of the data analysis are in accordance with those reported in the literature [15], indicating that the assumed sitting postures, along with the transitions between them and the overall seated mobility are associated with engagement as perceived by the player. Associations pointing in that direction were identified in both investigated types of observation frames. In continuous frames, the significant increase observed in the mean value (μA) and area (∫A) of the annotation trace from Tutorial to Gameplay was accompanied by a significant shift in assumed positions. The percentage of postures that include laying on the back of the chair was significantly lower in Gameplay than in the Tutorial, with many participants leaning forward as engagement increased and game interaction intensified. This shift was also present on overall mobility (μT) during continuous observation frames, but no statistical significance was observed. From Gameplay to Review, sitting postures activating the back of the chair appeared to increase in frequency, accompanied by a decrease in perceived engagement (μA and ∫A). However, these changes in assumed postures were not found to be statistically important. In contrast, a significant increase in perceived engagement from Review to Mission-2 was not accompanied by a significant change in identified postures. This may be in part because the Review has rather short duration, in comparison to the Tutorial and Gameplay. Additionally, the observed increase in perceived engagement between Review and Mission-2 was smaller than the one from Tutorial to Gameplay. These findings were consistently present in reactive observation frames. The investigated in-game events, for both 10 s and 30 s frame duration, resulted in a significant increase in three extracted features (μA, ∫A and $\hat{A}$) of the annotation trace of perceived engagement. Furthermore, the frequency of postures activating the back of the chair was lower in reactive frames following in-game events, being of statistical significance only for the case of 30 s reactive frames. Finally, an increase in overall player mobility (μT) was also evident after in-game events, but no statistical significance was observed. 

These findings indicate that an increase in perceived engagement is accompanied by the tendency to leave the back of the chair and an increased overall seated mobility (μT). The significance of these observations appears to be affected by the duration of the observation frames, with increased time yielding more significant results in both continuous and reactive frames. These observations are in agreement with findings of other studies that have employed different types of interaction tools [15,50]. The presented results can be a stepping-stone for the development of systems for real time recognition of engagement during SG play in office and home settings. In this direction, the identification of a general sensor activation threshold for posture monitoring, as proposed in the present study, is important. To this end, further investigation is necessary regarding the robustness of the threshold activation threshold across the BMI spectrum. Additionally, postures observed and identified during the intervention were not particularly relaxed. Even though the participants were instructed to get comfortable, this appears to have been hindered by their presence in a research setting. This is expected to affect the type and number of postures assumed in other settings. A single and quite standard office chair was employed during the present study. Desks in home settings tend to include chairs that vary greatly in size and comfort. The importance of data collection from home settings is, thus, highlighted and expected to provide more reliable results. 

The second part of the analysis has identified significant predictive capacity of both sensor-based sources and in-game metrics towards player perceived engagement, as reflected by their significant correlation with features from the annotation trace (amplitude ($\hat{A}$), mean value (μA), and area (∫A)). No significant correlation with the average gradient (ΔA) of the annotation trace has been found, despite its reported efficiency and robustness [39]. This may be attributed to the different data sources employed in this study and the larger duration of the examined observation frames. Reactive frames appear to produce the majority of features with significant predictive value, with 30 s frames revealing the most significant correlations. Features from sensors and in-game metrics present a range of significant and highly significant correlations (absolute values in the range 0.26–0.51) with perceived engagement, across all types of observation frames. However, an overly superior, in terms of robustness and effectiveness, unimodal feature could not be identified. On the contrary, the generated multimodal feature (*V*) was consistently found to be significantly correlated with features of the annotation trace. 

In summary, data collected from affordable and unobtrusive sensors, assisted by in-game metrics features, appear to hold predictive value towards the hypothesized ground truth. Nevertheless, the presented analysis has investigated these features’ predictive capability in a linear fashion. Supervised ML techniques can be employed to assess the features’ potential to accurately recognize engagement in a non-linear fashion. Such methods can be incorporated in PCG, as part of a constant feedback loop that enhances adherence to SG-based health interventions by maximizing player engagement through generated game content. The suitability of the multimodal feature *V* needs to be further validated through advanced feature fusion techniques via ML. Deep learning methodologies, along with larger datasets, can be employed in this investigation, given their increasing popularity in the field of multimodal affective recognition. Issues related to inclusivity and gender representation in participants should also be investigated for the purpose of identifying the potential impact of dataset imbalances on our core findings.

## 5. Conclusions

The present study investigates the feasibility of sensor-based real-time recognition of engagement during play in SGs for health. Multimodal data collected from pressure and heart rate sensors, as well as through participants’ interaction with the SG (in-game metrics) have been analyzed with the aim of identifying important correlations with the perceived engagement. The ground truth has been approximated by continuous annotation of perceived engagement by the participants. Sufficiently short observation frames based on player-generated in-game events and the type of game interaction provided the basis for a detailed data analysis which revealed (i) significant associations between identified postures and perceived engagement, and (ii) significant correlations of sensor and in-game metrics features with the hypothesized ground truth, both in unimodal and multimodal fashion, thus, highlighting their predictive capability in real-time. The robustness and efficiency of a generated multimodal feature (*V*) points towards the advantages of multimodal approaches in the recognition of engagement. These findings support future development of PCG techniques incorporating real-time engagement feedback loops that can significantly enhance SG health interventions, by maximizing engagement and increasing adherence.

## Figures and Tables

**Table 1:** Set of sitting postures and their activation patterns.

Posture	Description	Activated Sensors	Sensor Location on Chair
P1	Upright position with backrest	(3 or 7) and (4 or 8) and (a or d) and (b or c)	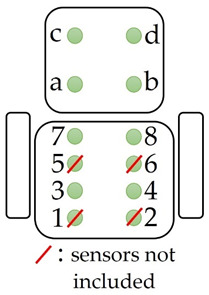
P2	Upright position without backrest	(3 or 7) and (2 or d)	
P3	Front sitting position	(3 or 4)	
P4	Front sitting position with backrest	(3 or 4) and (a or b or c or d)	
P5	Upright position with right backrest	(3 or 7) and (2 or 8) and (a or c)	
P6	Upright position with left backrest	(3 or 7) and (2 or 8) and (b or d)	

**Table 2:** Number of pairwise comparisons included in different types of observation frames.

	Reactive Frames (10 s)	Reactive Frames (30 s)	Continuous Frames
Primary features	76 ± 5.6	83.7 ± 3.7	56.4 ± 0.6
Multimodal feature	76.5 ± 1.1	82.7 ± 0.4	55.7 ± 0.4

**Table 3:** Correlation of annotation features and features extracted from posture sensors, heart rate sensor, and in-game metrics. Significant values (*p* < 0.05) are depicted in bold. Highly significant values (*p* < 0.01) are denoted by (*).

Annotation Features	Reactive Frames (10 s Duration)						Reactive Frames (30 s Duration)						Continuous Frames					
	μMc	μMm	**Δ** ***T***	$\hat{H}$	**Δ** ***H***	V	μMc	μMm	**Δ** ***T***	$\hat{H}$	σH	V	μMc	μMm.	**Δ** ***T***	$\hat{H}$	σH	V
μA	0.00	0.20	0.34 *	0.00	0.12	**0.26**	**0.28**	**0.51** *	0.01	0.01	−0.12	**0.25**	-	-	−0.05	−0.11	−0.05	−0.25
∫A	−0.01	0.19	0.33 *	0.01	0.14	**0.27**	0.30 *	**0.49** *	0.03	0.04	−0.10	**0.28**	-	-	−0.05	−0.11	−0.05	−0.25
$\hat{A}$	**−0.29**	0.13	0.00	0.06	**−0.33** *	−0.13	−0.05	**0.31** *	**0.26**	**0.24**	0.08	**0.33** *	-	-	0.25	0.42 *	0.18	**0.42** *
ΔA	−0.07	0.04	0.01	0.16	−0.11	0.15	0.06	0.18	0.11	0.13	−0.21	0.07	-	-	−0.12	0.14	−0.26	−0.07

## Data Availability

The data presented in this study are available on request from the corresponding author, in accordance with consent provided by the authors to researchers working on the field.
